# What is the relationship between family closeness, social support, and adolescent physical activity? Evidence from longitudinal tracking

**DOI:** 10.3389/fpsyg.2025.1718686

**Published:** 2025-12-09

**Authors:** Hanyu Li, Xielin Zhou, Wanbin Yu

**Affiliations:** Chengdu Sport University, Chengdu, Sichuan, China

**Keywords:** family closeness, social support, physical activity, adolescents, cross-lagged effects

## Abstract

**Purpose:**

Using a longitudinal cross-lagged design, this study examines the intrinsic relationship between family closeness, social support, and adolescent physical activity.

**Methods:**

Using the Family Intimacy Questionnaire, Social Support Rating Scale (SSRS), and Physical Activity Rating Scale (PARS), a two-phase longitudinal study was conducted on 802 adolescents (52.5% female) with a three-month interval between assessments.

**Results:**

(1) For adolescents, significant gender differences were found in family closeness, social support, and physical activity (*p* < 0.01); (2) Correlation analysis revealed positive pairwise correlations among family closeness, social support, and physical activity (all *p* < 0.01); (3) Cross-lagged analysis indicated that T1 family closeness significantly and positively predicted T2 social support (*β* = 0.30, *p* < 0.01), while T1 social support also significantly and positively predicted T2 family closeness (*β* = 0.30, *p* < 0.01). Furthermore, T1 family closeness significantly and positively predicted T2 physical activity (*β* = 0.29, *p* < 0.01), and T1 physical activity significantly and positively predicted T2 family closeness (*β* = 0.17, *p* < 0.01); T1 social support significantly and positively predicted T2 physical activity (*β* = 0.36, *p* < 0.01), and T1 physical activity significantly and positively predicted T2 social support (*β* = 0.22, *p* < 0.01).

**Conclusion:**

(1) Significant gender differences exist in family closeness, social support, and physical activity among children and adolescents; (2) Family closeness and social support are positively correlated with adolescent physical activity; (3) Within the adolescent cohort, family closeness and social support cross-predict physical activity over time, while social support and physical activity also cross-predict each other over time.

## 1 Introduction

Regular physical activity supports the development of the cardiovascular system, reduces the likelihood of disease occurrence, and promotes lifelong participation in sports (Karademir et al., 2024). From the 2015 Guiding Opinions on Strengthening Family Education, which first proposed “establishing a modern education system that organically integrates school education, family education, and social education,” to the 2023 Opinions on Improving the Mechanism for Collaborative Education Among Schools, Families, and Society, which continues to refine the responsibilities and coordination pathways for collaborative education among schools, families, and society (China, C. P., 2022). By 2024, the “School-Home-Community Collaborative Education ‘Teaching Consortium’ Work Plan” aims to promote effective collaboration among families, schools, and communities. This initiative centers on schools as hubs, leverages regional resources as connecting links, and prioritizes the healthy and joyful development of primary and secondary students as its core objective (Education, M. O., 2024). The evolution of these policies demonstrates that youth sports and physical and mental health have been elevated to a key agenda in social development, forming a multi-stakeholder consensus involving families, society, and schools. However, the tension between policy aspirations and social realities persists. Results from China’s Student Physical Fitness Monitoring indicate that while overall indicators show improvement, structural issues such as myopia, overweight/obesity, and declining physical fitness remain prominent (Network C. W., 2021). This phenomenon inevitably prompts reflection: why does youth physical inactivity persist as a persistent gap despite clear policy direction and a gradually forming social consensus? Clarifying this issue not only provides evidence-based grounds for targeted interventions but also advances the goal of “building robust student physical fitness” and promotes societal health development.

## 2 Literature review and problem statement

Physical activity (PA) is a key pathway for promoting the physical and mental health development of children and adolescents (Lemberg et al., 2021). Ecosystem theory suggests that PA is not merely an individual behavior, but is embedded within a multi-layered interaction involving family, school, and community (Li et al., 2025). Among these, the family, as the primary socialization setting for individuals, is widely regarded by scholars as a key variable influencing PA, with its internal emotional cohesion—that is, family intimacy—being a critical factor (Day, 2025). Family closeness refers to the emotional, psychological, and behavioral bond among family members, reflecting the degree of mutual care, support, and connection between them. Research by Chinese scholars indicates that close family relationships contribute to higher levels of physical activity among college students at home (Hanping, 2021). Xu Lulu and Dong Baolin also noted that parental support positively influences adolescents’ attitudes toward sports (Lulu and Baolin, 2021).

However, from a theoretical perspective, the relationship between family intimacy and physical activity has not yielded consistent findings across studies in China and other countries. For example, in China, family closeness significantly promotes physical activity among adolescents. This may be related to the emphasis on family bonds and collectivist values in Chinese culture, where strong ties and mutual support among family members provide a solid emotional foundation and motivational support for adolescents’ participation in physical activities (Kuo et al., 2007). However, in other countries, such as the United States and other Western nations, adolescents’ participation in physical exercise tends to be more casual. Their primary motivations for engaging in exercise include improving health, seeking recreation, and weight loss (Li, 2025), with weaker correlations observed between exercise participation and family closeness. This may be related to the emphasis on individualism and personal autonomy in Western culture, where adolescents are more inclined to choose whether to participate in physical activities based on their own interests and needs. The differing conclusions from previous studies raise questions: What is the intrinsic relationship and underlying logic between family closeness and physical activity among adolescents? Clearly, further empirical research is needed to explore this topic.

In recent years, the concept of “collaborative education among families, schools, and communities” has gained widespread acceptance. As a key component of this approach, social support not only provides crucial backing for family and school educational efforts but also fosters an external environment conducive to promoting the physical and mental well-being of adolescents. Social support refers to the emotional or material assistance individuals provide to one another, playing a vital role in the healthy physical and mental development of people (Frisch et al., 2014). Research indicates that social support plays a particularly crucial role in adolescents’ physical exercise routines, with the social support individuals receive contributing to enhanced exercise persistence (Sarkar et al., 2016). From an international research perspective, Western countries have taken an early lead in studying the relationship between social support and adolescent physical activity, yielding abundant findings. For instance, in European and American countries, well-developed community sports facilities and diverse physical activities organized by schools and communities provide adolescents with multifaceted social support from families, schools, and communities (Davis et al., 2021). This support encompasses not only material resources such as sports equipment and venues but also emotional and informational support, including parental encouragement, coaching guidance, and peer companionship. The combined effect of these multidimensional social supports effectively promotes adolescents’ participation in and persistence with physical activity. Furthermore, as a social behavior, physical activity’s inherent social characteristics also influence an individual’s social support. Cross-sectional studies reveal that exercise positively impacts adolescents’ social support, with physical activity levels showing a positive correlation with social support scores (Shu et al., 2024). Specifically, good exercise habits enhance participants’ communication frequency, thereby cultivating their emotional, moral, and character development. This influences their social support networks, serving as a unique vehicle for developing social–emotional competencies. Thus, it is evident that social support and physical activity may be interconnected.

Furthermore, sociocultural theory emphasizes that individual development is embedded within a multi-level environmental system. As a micro-system within this framework, the family’s internal emotional bonds—that is, family intimacy—play a foundational role in an individual’s social development. This theory posits that high family intimacy provides individuals with intrinsic psychological resources for establishing and maintaining external social support networks by shaping their cognitive patterns, value systems, and social interaction abilities (Zhu Nan and Zou, 2015). Therefore, from a sociocultural perspective, family intimacy is regarded as a crucial precursor to social support, with its influence pathway summarized as “from the inside out.” That is, family closeness may predict an individual’s level of social support. However, Social Support Theory (SST) and Family Capital Theory (FCT) propose a counterargument from the perspective of resource input and family system functioning, suggesting that social support may predict family intimacy in the opposite direction. SST indicates that the emotional and instrumental support individuals receive from social networks can effectively buffer external stressors, enhancing psychological well-being and family functioning (Van Petegem et al., 2018). This improvement manifests specifically in enhanced communication quality and strengthened emotional bonds among family members, thereby reinforcing family intimacy (Barzoki and Toikko, 2025). FCT further views social support as a crucial form of capital external to the family, positing that the infusion of such capital enriches the family’s internal resource pool, optimizes family functioning, and thereby consolidates and enhances family intimacy (Wei Lingzhen and Liu, 2021). Thus, SST and FCT jointly construct an “outside-in” pathway, wherein social support positively predicts changes in family intimacy (Qiu and Wang, 2025). Although previous cross-sectional studies have also examined the relationship between the two, some scholars suggest that family closeness and adaptive functioning are positively correlated with social support. At the same time, other researchers have found that social support can influence family closeness and sense of meaning in life (Campos et al., 2014). However, the longitudinal mechanisms linking family closeness and social support among adolescents require further investigation.

**Figure 1:**
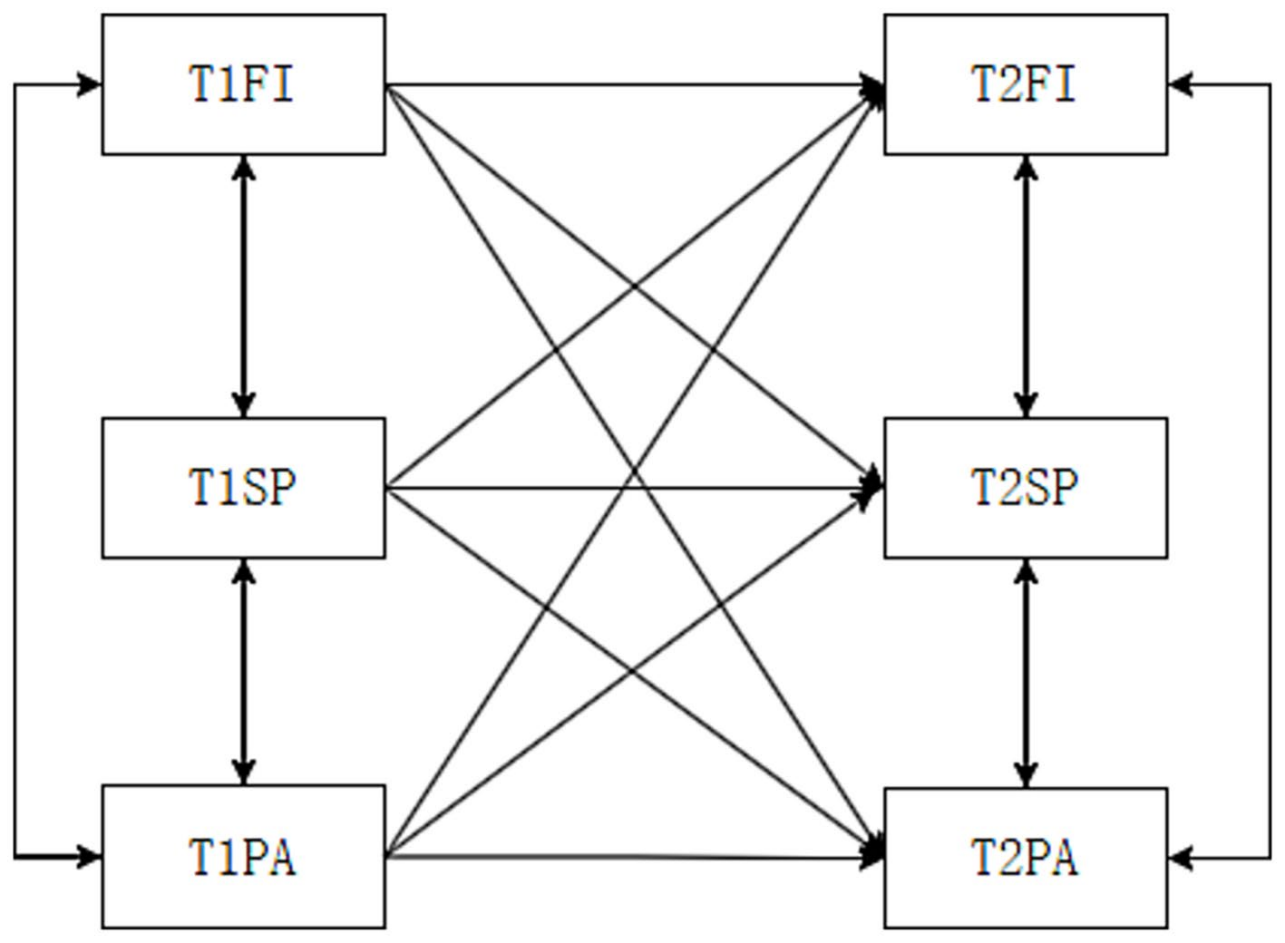
Cross-lagged structural equation model diagram.

In summary, although previous studies have explored the mechanisms underlying physical activity and achieved some progress, what are the longitudinal associations between family closeness, social support, and physical activity among adolescents? What roles do family closeness and social support play in the context of adolescent physical activity? Are there gender differences in the relationship between family closeness, social support, and physical activity among children and adolescents? Given the limitations of cross-sectional studies in terms of stability, this study employs a longitudinal design with a cross-lagged approach, using structural equation modeling to examine the intrinsic relationships and mechanisms among family closeness, social support, and adolescent physical activity. It hypothesizes a predictive relationship among these three factors, as illustrated in Figure 1.

## 3 Materials and methods

### 3.1 Participants

This study selected adolescents aged 12–18 (junior high and high school students) and followed stratified sampling principles to select 20 secondary schools in Southwest China. Five schools were chosen from each of Sichuan Province, Chongqing Municipality, Guizhou Province, and Yunnan Province. One class per grade level was randomly selected from each school as the sampling unit. Considering the concentrated nature of adolescents’ physical activity during school hours, a three-month longitudinal tracking survey was conducted from September to December.

Prior to administering the questionnaire, the G*Power program was used to calculate the minimum sample size. With *α* = 0.05, *β* = 0.80, and Effect Size = 0.15, the minimum sample size required for the study was determined to be 298. Subsequently, the homeroom teacher was informed of the questionnaire administration process. After being entrusted to explain the purpose of this study to participants and assure them that questionnaire information would be kept strictly confidential, participants completed and returned the questionnaires within the specified timeframe. The study was conducted at two time points, with 1,000 questionnaires distributed at each. The T1 measurement was administered offline from September 1 to 15, 2024. Based on screening criteria such as “missing information” and “reverse item testing,” 139 invalid questionnaires were excluded, yielding 861 valid responses at T1. Measurements for T2 were conducted from December 1 to 15, 2024. Due to objective reasons such as leave of absence or illness among some participants, 823 valid questionnaires were ultimately collected. The final valid sample comprised 802 participants who completed questionnaires at both time points and who’s last five digits of student ID numbers matched, yielding an effectiveness rate of 80.2%. Among them, 381 were male, accounting for 47.5%, while 421 were female, accounting for 52.5%. The average age was 14.71 ± 1.02 years.

### 3.2 Methods

#### 3.2.1 Family intimacy questionnaire

The Family Intimacy and Adaptability Scale, developed by Fei and Zheng (1991), was selected to evaluate adolescents’ family relationships (Fei and Zheng, 1991). This scale comprises 16 items across two dimensions—intimacy and adaptability—and employs a 5-point Likert scale (1 = strongly disagree, 5 = strongly agree). Items 13–16 are reverse-scored. In this study, the Cronbach’s alpha coefficients at time points T1 and T2 were 0.948 and 0.949, respectively. The confirmatory factor analysis results showed: $\chi^{2}/\mathrm{df}$ = 1.028, GFI = 0.983, CFI = 0.998, IFI = 0.998, TLI = 0.998, RMSEA = 0.006 (Test1); $\chi^{2}/\mathrm{df}$ =1.052, GFI = 0.9883, CFI = 0.999, IFI = 0.999, TLI = 0.999, RMSEA = 0.008 (Test2), indicating that the scale possesses good reliability and validity.

#### 3.2.2 Social support rating scale

The Social Support Rating Scale, developed by Ye Yuemei and Dai Xiaoyang, was employed (Yuemei and Xiaoyang, 2008). Based on Xiao Shuiyuan’s theoretical model of social support, this 17-item scale assesses three factors: subjective support, objective support, and support utilization. It utilizes a 5-point Likert scale (1 = strongly disagree, 5 = strongly agree), with higher scores indicating greater levels of social support. In this study, the Cronbach’s alpha coefficients at time points T1 and T2 were 0.926 and 0.900, respectively. The confirmatory factor analysis results showed: $\chi^{2}/\mathrm{df}$ = 2.116, GFI = 0.968, CFI = 0.983, IFI = 0.983, TLI = 0.980, RMSEA = 0.037 (Test1); $\chi^{2}/\mathrm{df}$ =2.080, GFI = 0.967, CFI = 0.983, IFI = 0.983, TLI = 0.980, RMSEA = 0.037 (Test2). These findings indicate that the scale possesses good reliability and validity.

#### 3.2.3 Physical activity level scale

The Physical Activity Level Scale developed by Qing (1994) was adopted. This scale, used by domestic scholars to measure physical activity among children and adolescents, comprises three dimensions: exercise intensity, duration, and frequency. It employs a 5-point Likert scale, with the total physical activity score calculated as the product of the scores across these three dimensions. Activity levels are categorized as low intensity (0–19 points), moderate physical activity (20–42 points), and high physical activity (≥43 points) (Qing, 1994). The Cronbach’s alpha coefficients at time points T1 and T2 were 0.814 and 0.819, respectively. The exploratory factor analysis results showed: KMO coefficient of 0.670, Bartlett’s test of sphericity *p* < 0.001, and principal components explaining 73.311% of variance (Test1); KMO coefficient of 0.607, Bartlett’s test of sphericity p < 0.001, and 74.084% variance explained by principal components (Test2). These findings indicate that the scale possesses good reliability and validity.

#### 3.2.4 Statistical methods and data analysis

This study employed SPSS 27.0 and AMOS 26.0 to process and analyze the collected data. SPSS was utilized for examining common method bias, descriptive statistics, correlation analysis, independent samples t-tests, analysis of variance, and confirmatory factor analysis. AMOS was applied for model construction and variable examination, testing the model’s autoregressive coefficients, factor correlation path coefficients, and cross-lagged path coefficients. Model fit was assessed using the Maximum Likelihood method.

### 3.3 Common method bias test

This study employed Harman’s one-factor test to examine common method variance (Zhou Hao, 2004). At the first time point (T1), five factors with eigenvalues greater than 1 were extracted, with the first factor cumulatively explaining 35.332% of the total variance. The results at the second time point (T2) extracted five factors with eigenvalues greater than 1. Among these, the first factor cumulatively explained 35.339% of the total variance. At both time points, the values were below the 40% critical threshold, indicating that neither the T1 nor T2 measurements in this study exhibited severe common method bias.

## 4 Results

### 4.1 Descriptive statistics and correlation analysis of family intimacy, social support, and adolescent physical activity

In this study, descriptive statistics and correlation analyses were conducted on three variables: family closeness, social support, and physical activity. The results are presented in Table 1. Correlation analysis revealed that at both T1 and T2, family closeness scores, social support scores, and physical activity scores exhibited significant positive correlations (*p* < 0.01). Family closeness scores at the two time points showed a significant positive correlation (*p* < 0.01); social support scores at the two time points showed a significant positive correlation (*p* < 0.01); physical exercise behavior scores at the two time points showed a significant positive correlation (*p* < 0.01), satisfying the conditions for constructing a cross-lagged model. Specifically, T1 family closeness showed significant positive correlations with T1 social support (*r* = 0.442), T1 physical activity (*r* = 0.375), T2 social support (*r* = 0.522), and T2 physical activity (*r* = 0.517; *p* < 0.01); T1 social support showed significant positive correlations with T1 physical activity (*r* = 0.405), T2 family closeness (*r* = 0.473), and T2 physical activity (*r* = 0.559; *p* < 0.01); T1 physical activity showed significant positive correlations with T2 family closeness (*r* = 0.368) and T2 social support (*r* = 0.442; *p* < 0.01); T2 family closeness showed significant positive correlations with T2 social support (*r* = 0.472) and T2 physical activity (*r* = 0.500; *p* < 0.01); T2 social support also showed a significant positive correlation with T2 physical activity (*r* = 0.647, *p* < 0.01).

**Table 1:** Mean, standard deviation and correlation coefficient between variables.

	T1FI	T1SP	T1PA	T2FI	T2SP	T2PA
T1FI	1					
T1SP	0.442**	1				
T1PA	0.375**	0.405**	1			
T2FI	0.429**	0.473**	0.368**	1		
T2SP	0.522**	0.528**	0.442**	0.472**	1	
T2PA	0.517**	0.559**	0.433**	0.500**	0.647**	1
Mean						
SD						

“*” indicates *p* < 0.05, “**” indicates *p* < 0.01.

### 4.2 Analysis of gender differences in family intimacy, social support, and adolescent physical activity

According to the central limit theorem, in psychological research, a sample size greater than 200 can be considered to satisfy a normal distribution (Armstrong et al., 2019). In this study, an independent samples t-test was conducted on participants’ gender. Significant gender differences were found across all dimensions (all *p* < 0.01). This indicates variations in family closeness, social support, and physical activity based on gender. Further analysis revealed that the t-test results showed males scored higher than females on all dimensions (Table 2).

**Table 2:** Gender differences in variables.

	Male	Female	t	*p*
T1FI	3.02 ± 0.88	2.69 ± 0.77	5.59	<0.01
T1SP	3.09 ± 0.83	2.66 ± 0.67	8.07	<0.01
T1PA	3.11 ± 1.11	2.65 ± 0.91	6.34	<0.01
T2FI	3.02 ± 0.89	2.61 ± 0.76	7.09	<0.01
T2SP	3.06 ± 0.82	2.70 ± 0.58	7.11	<0.01
T2PA	3.09 ± 1.08	2.56 ± 0.83	7.68	<0.01

### 4.3 Cross-lagged analysis of family intimacy, social support, and adolescent physical activity

**Figure 2:**
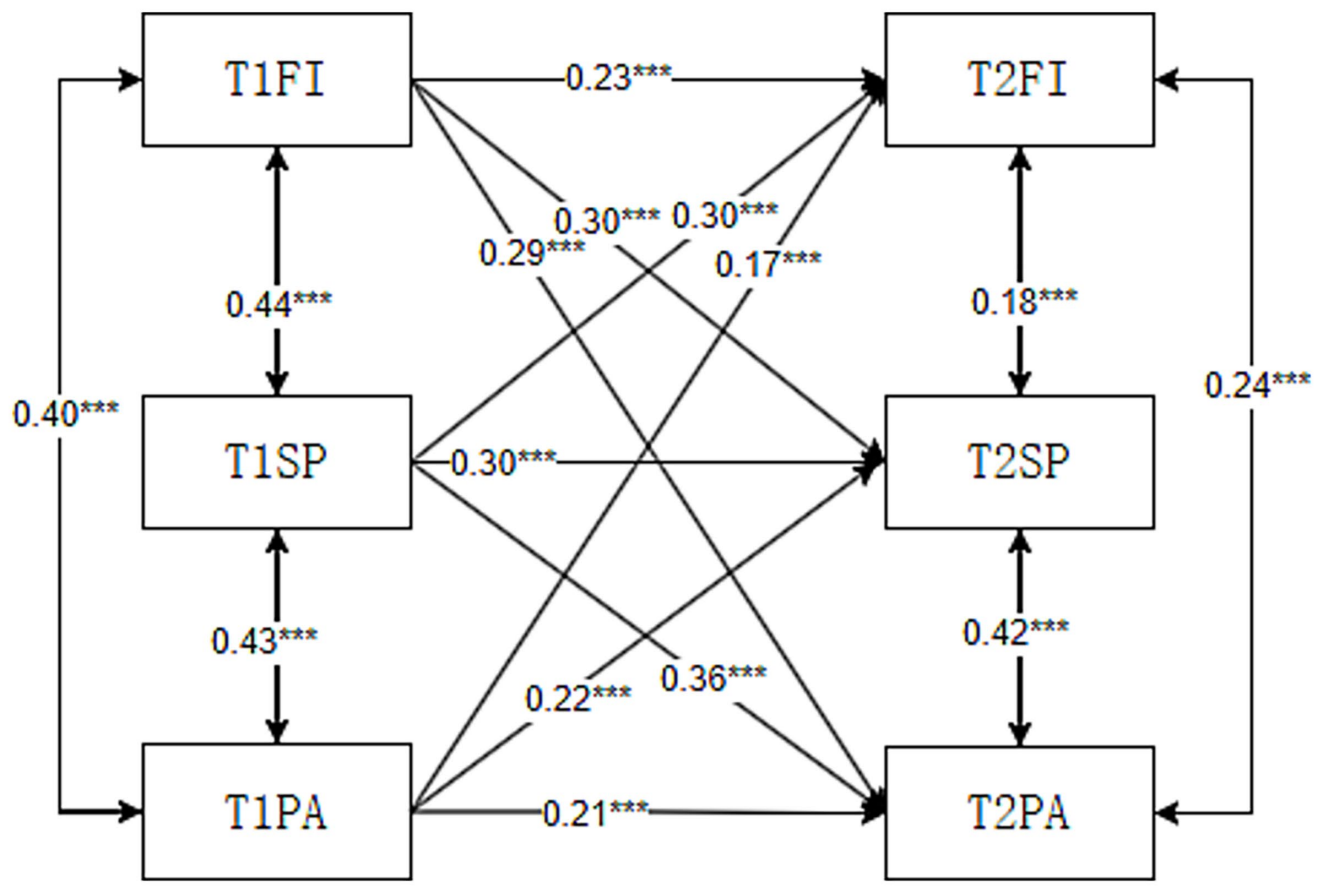
Cross-lagged model diagram.

Using AMOS 26.0, we constructed the first model, M1, of this study: a structural equation model examining family closeness, social support, and adolescent physical exercise behavior. Employing maximum likelihood estimation, the model fit indices showed $\chi^{2}/\mathrm{df}$ = 1.626, GFI = 0.927, CFI = 0.973, IFI = 0.973, TLI = 0.971, RMSEA = 0.028, indicating good model fit (Table 3). Simultaneously, path value observations revealed that T1 family closeness significantly and positively predicted T2 social support (*β* = 0.30, *p* < 0.01), while T1 social support also significantly and positively predicted T2 family closeness (*β* = 0.30, *p* < 0.01). Furthermore, T1 family closeness significantly and positively predicted T2 physical activity (*β* = 0.29, *p* < 0.01), and T1 physical activity significantly and positively predicted T2 family closeness (*β* = 0.17, *p* < 0.01). T1 social support significantly and positively predicted T2 physical activity (*β* = 0.36, *p* < 0.01), and T1 physical activity predicted T2 social support (*β* = 0.22, *p* < 0.01; Figure 2). It can thus be seen that family closeness and social support predict each other, family closeness and physical activity predict each other, and social support and physical activity predict each other.

**Table 3:** Indicators of model fit.

Model name	$\chi^{2}/\mathrm{df}$	GFI	CFI	IFI	TLI	RMSEA
M1	1.626	0.927	0.973	0.973	0.971	0.028
Standard	<3	>0.900	>0.900	>0.900	>0.900	<0.08

## 5 Discussion

### 5.1 Analysis of gender differences in family intimacy, social support, and physical activity among adolescents

This study found significant gender differences in scores for family closeness, social support, and physical activity across two measurements. Male adolescents scored higher than females on all three variables, indicating that gender is an important demographic factor explaining differences in adolescent health behaviors. Within the family closeness dimension, gender role differences were observed, consistent with recent research findings in East Asian cultural contexts (Shao and Ni, 2021). In Chinese families, parents’ dual expectations for boys—achievement and health—often materialize through “accompanied support.” For instance, fathers are more inclined to join their sons in activities like sports training camps and outdoor hikes, fostering high-quality interactions through shared “physical challenges.” Girls, however, are assigned more “academic-reserved” role expectations. Parent–child interactions primarily revolve around homework assistance and home conversations. While emotional exchanges are frequent, there is a lack of synchronized physical activities. This results in high “emotional warmth” between parent and child, yet it struggles to translate into a virtuous cycle of “intimacy-physical activity.”

In terms of social support, males scored higher than females. On one hand, males more readily access dual “tool-information” support: within peer groups, athletic ability remains a key source of social prestige for boys. Through team sports like basketball and soccer, males gain group recognition, forming a reinforcing loop of “sports → peer affirmation → more sports.” Girls, however, face higher barriers to participation due to “body image anxiety” and “gender stigma.” Peer discourse centered on “weight loss rather than competition” diminishes the social function of physical activities. On the other hand, research has found that physical education teachers provide more frequent feedback to male students than to female students in classroom questioning and the allocation of demonstration opportunities (Leisterer and Paschold, 2022). This disparity in information access further widens the gender gap in social support.

In the physical activity dimension, males reported significantly higher levels than females, a finding consistent with numerous studies worldwide (DeWolfe et al., 2020). This disparity stems from the combined influence of biological and physiological factors and sociocultural constructs. From a biological perspective, adolescent males typically possess greater muscle mass and higher testosterone levels than females, which may fundamentally contribute to their higher energy levels and greater inclination toward moderate-to-high intensity physical activities. However, from a sociocultural perspective, the realm of sports has long been constructed as a key arena for demonstrating masculinity, competitiveness, and strength. Society expects boys to be “active” and “strong,” making sports activities one of the pathways through which they conform to gender role norms and establish social identity (Jackson et al., 2022). Conversely, for adolescent girls, societal beauty standards often emphasize “graceful” and “slender” over “strong and muscular.” This can lead to body image anxiety, as they may fear being labeled “masculine” or worry that exercise will result in an “unattractive” physique. Consequently, they may actively avoid certain activities—particularly strenuous ones that cause heavy sweating.

In summary, the gender difference analysis findings of this study indicate that adolescent physical activity levels should not be viewed in isolation. Instead, they should be understood within a holistic ecological framework that encompasses family systems and social networks, all of which are mediated by gender role norms. Therefore, future interventions to promote physical activity among adolescents—particularly girls—should not only encourage individual behavioral change but also focus on transforming gendered interaction patterns within families. This requires fostering a more supportive and encouraging social environment for girls, challenging traditional stereotypes, and creating equitable, positive conditions for physical participation for all adolescents.

### 5.2 The relationship between family intimacy, social support, and adolescent physical activity

This study systematically examined the predictive relationships among family closeness, social support, and adolescents’ physical activity at school using a cross-lagged model based on two longitudinal follow-up datasets. Findings indicate that, overall, family closeness exerts a stable influence across time on both social support and adolescents’ physical activity; social support and physical activity at school exhibit a mutually predictive pattern.

First, family closeness can positively predict social support, consistent with previous research findings (Markowski et al., 2023). This finding indicates that adolescents who experience greater intimacy within their families are more likely to receive increased social support in the future. As the primary setting for adolescent socialization, a close-knit family environment fosters positive social patterns and interpersonal trust, thereby facilitating access to social support from peers, teachers, and others. Furthermore, social support positively influences family intimacy. On one hand, adolescents with higher levels of social support can promptly regulate negative emotions when facing setbacks, thereby enhancing their mental well-being. Social exchange theory further substantiates this perspective, positing that individuals tend to reciprocate favors after receiving benefits. Since the family serves as adolescents’ primary emotional attachment, reciprocity often manifests as increased self-disclosure toward parents, greater compliance, and proactive initiation of intimate interactions (such as sharing accomplishments or inviting parents to events), thereby enhancing emotional warmth between parent and child (Yu et al., 2022).

Furthermore, family closeness can positively predict physical activity, consistent with previous scholarly views (Senguttuvan et al., 2014). This study further enriches longitudinal tracking evidence in this field. Findings indicate that adolescent physical activity is influenced by intergenerational relationships, individual differences, and social environments. As the starting point for individual development, the family serves both as the origin of behavioral habits and a crucial arena for growth. As family systems theory suggests, parents exert significant influence on their children’s development (Chen and Liu, 2023). Adolescents from families with high intimacy levels benefit from home environments that foster healthier lifestyle habits and behavioral patterns. Close interactions among family members—such as engaging in physical exercise and outdoor activities together—not only boost adolescents’ interest and participation in sports but also provide essential material and emotional support, encouraging sustained physical activity. Meanwhile, prior research suggests that college students’ exercise habits cannot be predicted by family relationships (Zhou et al., 2024). However, this study found that physical activity also positively predicts family closeness among adolescents, thereby enriching previous research perspectives. This may be because adolescents remain in the family-centered phase, with daily activities highly dependent on parental provision of time, financial support, and transportation. College students, however, have entered an individualized stage where their living environments and decision-making authority are largely detached from the family. Consequently, the same “physical activity” cues are more readily captured by parents in adolescent households and transformed into shared topics or opportunities for companionship, thereby reinforcing familial closeness.

Finally, this study found that social support positively predicts physical activity, while physical activity also positively predicts social support. This result enriches the previous unidirectional “support → behavior” relationship, further revealing the intrinsic connection between social support and physical activity. Building upon the “support → behavior” framework, research indicates that adolescents’ physical activity is influenced by multiple factors, including family, society, and school, with social support emerging as a significant component (Xiao and Tang, 2025). Support from peers and teachers provides the initial impetus for adolescents to engage in physical activity, lowering barriers to participation and enhancing motivation. Furthermore, from a “behavior → support” perspective, active involvement in physical activities—particularly team sports—is regarded as a crucial arena for adolescents to build social capital. Within this setting, they actively shape their social support networks by forging friendships, strengthening relationships, demonstrating abilities, and gaining recognition through practice (Hamilton and White, 2010). Previous studies have also indicated that the more frequently individuals participate in physical activities, the greater the social support they receive in daily life.

### 5.3 Research strengths and limitations

This study reveals the intrinsic relationship between family closeness, social support, and adolescent physical activity, precisely focusing on the critical issues of “family,” “society,” and adolescent health in the present context. In terms of research design, this study employs a longitudinal tracking approach, effectively addressing the limitations of previous cross-sectional studies where results were susceptible to transient factors and lacked stability. From a practical application perspective, this research enables parents, teachers, and other stakeholders to gain deeper insights into the mechanisms influencing adolescent physical activity, thereby facilitating the development of family exercise plans and physical education curricula. However, this study still has limitations, and future research could be enhanced in the following areas:

1. This study aims to examine the relationship between family closeness, social support, and adolescent physical activity. Therefore, only gender was considered as a demographic variable, though other uncontrolled variables may still exist. For instance, parental education level, family socioeconomic status (SES), parental exercise habits, accessibility of community sports facilities, urban–rural regional differences, and adolescent personality traits (such as extraversion and self-discipline) may simultaneously influence family closeness, social support, and physical activity, thereby introducing potential confounding effects on the estimated results. Future research should incorporate and control for these key covariates in the model or employ multilevel linear models to distinguish individual-level and contextual-level effects, thereby enhancing the precision and external validity of conclusions.
2. This study employs a cross-lagged panel model (CLPM) to examine longitudinal relationships between variables. While this model can estimate mutual predictive effects across time points, it fails to effectively disentangle the influence of within-individual variation from that of stable inter-individual traits. The Random Intercept Cross-Lagged Panel Model (RI-CLPM) enables the estimation of dynamic interaction mechanisms at the individual level with greater purity. This is achieved by incorporating random intercepts at the individual level, thereby controlling for stable characteristics that do not change over time, such as baseline personality traits and family background. Therefore, future research may employ the RI-CLPM to further distinguish between stable inter-individual differences and intra-individual temporal variations, thereby more precisely elucidating the causal dynamics between family closeness, social support, and adolescent physical activity.
3. The follow-up period for this study spanned a total of 3 months. This design is appropriate and effective for capturing short-term interactions and initial effects between variables, making it suitable for examining the relationship between psychosocial factors and behavior. However, this timeframe is insufficient to fully reveal long-term, stable developmental trajectories among the variables. Therefore, future research may employ longer follow-up periods (such as 1 year or several years) and increase the frequency of measurement waves to examine the continuity and patterns of change in these relationships across different developmental stages of adolescence, thereby validating their dynamic patterns on a macro-developmental scale.

## 6 Conclusion

This study employed a cross-lagged design and concluded that: (1) Gender differences exist between family closeness and adolescents’ physical activity outside of school; (2) Family closeness and social support can predict each other across time; (3) Family closeness and adolescents’ physical activity predict each other across time; (4) Social support also predicts adolescents’ physical activity across time.

Based on the findings of this study, the following recommendations are proposed: (1) Governments, communities, schools, and families should recognize that enhancing adolescents’ physical fitness is a systemic endeavor. Policy development (e.g., Healthy Cities, Sunshine Sports) must consciously incorporate the strengthening of family relationships and social support networks as core indicators, rather than focusing solely on exercise duration or intensity. (2) Schools and communities should offer dual-menu sports programs—competitive activities for boys and rhythmic activities for girls—when establishing physical education initiatives. Implement a “gender quota” for classroom feedback by physical education teachers, ensuring girls receive at least as many skill-specific comments per lesson as boys to narrow initial skill gaps. (3) Establish a dual-mentor system of “teacher-peer” support during after-school programs: physical education teachers demonstrate skills while peers provide companionship and encouragement, creating immediate access to social support.

## Data Availability

The original contributions presented in the study are included in the article/supplementary material, further inquiries can be directed to the corresponding author.

## References

[ref1] Armstrong B. Covington L. B. Unick G. J. Black M. M. (2019). Featured article: bidirectional effects of sleep and sedentary behavior among toddlers: a dynamic multilevel modeling approach. J. Pediatr. Psychol. 44, 275–285. doi: 10.1093/jpepsy/jsy089, 30476202 PMC6551589

[ref2] Barzoki M. H. Toikko T. (2025). Family intimacy and depression: a comparative study among adolescents in Finland. Nord. J. Psychiatry 79, 70–78. doi: 10.1080/08039488.2024.2436986 39641266

[ref3] Campos B. Ullman J. B. Aguilera A. Schetter C. D. (2014). Familism and psychological health: the intervening role of closeness and social support. Cult. Divers. Ethn. Minor. Psychol. 20, 191–201. doi: 10.1037/a0034094, 24773004 PMC4170713

[ref4] Chen J. Liu C. H. (2023). The longitudinal association between children's growth mindset in senior primary school and their parents' growth mindset. Front. Psychol. 14:1110944. doi: 10.3389/fpsyg.2023.1110944, 36998367 PMC10043423

[ref5] China, C. P. (2022). Opinion of the Ministry of Education and 12 Other Departments on Improving the Mechanism for Collaborative Education Among Schools, Families, and Society. Available online at: https://www.gov.cn/zhengce/zhengceku/2023-01/19/content_5737973.htm (Accessed September 26, 2025).

[ref6] Davis A. J. MacCarron P. Cohen E. (2021). Social reward and support effects on exercise experiences and performance: evidence from parkrun. PLoS One 16. doi: 10.1371/journal.pone.0256546 PMC8443045 34525097

[ref7] Day J. (2025). The generational shift towards the reciprocal disclosure of intimacy in daughter-father relationships through physical activity in the UK. Families Relationships And Societies 14, 457–472. doi: 10.1332/20467435Y2024D000000052

[ref8] DeWolfe C. E. J. Watt M. C. Romero-Sanchiz P. Stewart S. H. (2020). Gender differences in physical activity are partially explained by anxiety sensitivity in post-secondary students. J. Am. Coll. Heal. 68, 219–222. doi: 10.1080/07448481.2018.1549048, 30645185

[ref9] Education, M. O. (2024). Work Plan for the "Educational Alliance" of Home-School-Community Collaborative Education. Available online at: http://www.moe.gov.cn/jyb_xwfb/gzdt_gzdt/s5987/202411/t20241101_1160204.html (Accessed September 27, 2025).

[ref10] Fei L. Zheng Y. (1991). Preliminary evaluation of the "family intimacy and adaptability scale" and "family environment scale": a comparative study of Normal families and families with schizophrenic member. Chin. J. Ment. Health 05, 198–202+238.

[ref11] Frisch J. U. Haeusser J. A. van Dick R. Mojzisch A. (2014). Making support work: the interplay between social support and social identity. J. Exp. Soc. Psychol. 55, 154–161. doi: 10.1016/j.jesp.2014.06.009

[ref12] Hamilton K. White K. M. (2010). Parental physical activity: exploring the role of social support. Am. J. Health Behav. 34, 573–584. doi: 10.5993/ajhb.34.5.7, 20524887

[ref13] Hanping L. (2021). The relationship among family intimacy, body self-esteem, and physical activity at home among college students. J. Tianjin Univ. Sport. 36, 563–568+589. doi: 10.13297/j.cnki.issn1005-0000.2021.05.010

[ref14] Jackson J. L. Fox K. R. Swenski T. N. Neville S. P. Marousis N. C. Korth C. X. . (2022). Gender differences in physical activity engagement among adolescents with congenital heart disease. J. Pediatr. Psychol. 47, 859–869. doi: 10.1093/jpepsy/jsab114, 34725688 PMC9608576

[ref15] Karademir A. Alkan H. Canli M. (2024). The effect of regular physical activities on neurological status, developmental parameters, and physical fitness in preschoolers. Early Child Dev. Care 194, 597–605. doi: 10.1080/03004430.2024.2343923

[ref16] Kuo J. Haythornthwaite J. A. Young D. R. (2007). Associations between family support, family intimacy, and neighborhood violence and physical activity in urban adolescent girls. Am. J. Public Health 91, 101–103. doi: 10.2105/AJPH.2005.072348 PMC1716242 17138926

[ref17] Leisterer S. Paschold E. (2022). Increased perceived autonomy-supportive teaching in physical education classes changes students' positive emotional perception compared to controlling teaching. Front. Psychol. 13:1015362. doi: 10.3389/fpsyg.2022.1015362, 36389480 PMC9665235

[ref18] Lemberg G. M. Kull M. Mägi K. Tilga H. Mooses K. Mäestu E. (2021). Higher physical activity of school personnel is related to more positive attitudes towards children's physical activity at school. Sustainability 13. doi: 10.3390/su131910909, 41291439

[ref19] Li X. Y. (2025). The relationship between quality of sports friendships and mental health in Chinese junior high school students: the bidirectional chain mediating effects of sport motivation and exercise adherence. BMC Public Health 25:52. doi: 10.1186/s12889-025-21287-5, 39762828 PMC11706195

[ref20] Li Y. Li S. S. Weng X. Yang X. Y. Bao J. Y. Liao S. F. . (2025). Effects of the Vivifrail-B multicomponent exercise program based on society ecosystems theory on physical function in community-dwelling frail older adults: a randomized controlled trial. Exp. Gerontol. 200:112670. doi: 10.1016/j.exger.2024.112670, 39736420

[ref21] Lulu X. Baolin D. (2021). The relationship between adolescents' perception of parental support and physical activity: a cross-lagged study. Chin. J. Sports Sci. 57, 60–66.

[ref22] Markowski K. L. Smith J. A. Gauthier G. R. Harcey S. R. (2023). Would I have your support? Family network features and past support exchanges associated with anticipated support for a substance problem. J. Subst. Abus. 28, 39–45. doi: 10.1080/14659891.2021.2006340, 36683732 PMC9856213

[ref23] Network C. W (2021). Overall improvement in students' physical fitness and health status persistent challenges in adolescent myopia and obesity. People's Information.

[ref24] Qing L. D. (1994). Stress level of college students and its relationship with physical exercise. Chin. J. Ment. Health 1, 5–6.

[ref25] Qiu J. Wang J. L. (2025). Influential factors of suicidal ideation among university students-the moderating role of family closeness and peer support. Int. J. Ment. Health Promot. 27, 485–505. doi: 10.32604/ijmhp.2025.059951

[ref26] Sarkar S. Taylor W. C. Lai D. J. Shegog R. Paxton R. J. (2016). Social support for physical activity: comparison of family, friends, and coworkers. Work 55, 893–899. doi: 10.3233/WOR-162459, 28059824

[ref27] Senguttuvan U. Whiteman S. D. Jensen A. C. (2014). Family relationships and adolescents' health attitudes and weight: the understudied role of sibling relationships. Fam. Relat. 63, 384–396. doi: 10.1111/fare.12073, 24954967 PMC4061754

[ref28] Shao X. Y. Ni X. L. (2021). How does family intimacy predict self-esteem in adolescents? Moderation of social media use based on gender difference. SAGE Open 11:21582440211005453. doi: 10.1177/21582440211005453

[ref29] Shu J. W. Chen Z. J. Zhong B. B. Ding Z. F. Tang S. H. Sun Z. C. . (2024). The relationship between physical activity and college students' perceived social support: the mediating role of social-emotional competence and its gender differences. J. Community Appl. Soc. Psychol. 34:e2835. doi: 10.1002/casp.2835

[ref30] Van Petegem S. Brenning K. Baudat S. Beyers W. Zimmer-Gembeck M. J. (2018). Intimacy development in late adolescence: longitudinal associations with perceived parental autonomy support and adolescents' self-worth. J. Adolesc. 65, 111–122. doi: 10.1016/j.adolescence.2018.03.008, 29573644

[ref31] Wei Lingzhen L. Y. Liu C. (2021). The effect of family intimacy on mental health among high school students: a mediated and moderated model. Psychol. Behav. Res. 19, 361–367.

[ref32] Xiao Q. Tang F. Y. (2025). Extracurricular physical exercise and self-education expectations among Chinese teenagers. Front. Psychol. 16:1518100. doi: 10.3389/fpsyg.2025.1518100, 40012943 PMC11862996

[ref33] Yu X. L. Kong X. T. Cao Z. Y. Chen Z. J. Zhang L. Yu B. B. (2022). Social support and family functioning during adolescence: a two-wave cross-lagged study. Int. J. Environ. Res. Public Health 19:6327. doi: 10.3390/ijerph19106327, 35627864 PMC9140348

[ref34] Yuemei Y. Xiaoyang D. (2008). Development of a social support assessment scale for college students. Chin. J. Clin. Psychol. 5, 456–458.

[ref35] Zhou Hao L. L. (2004). Statistical tests and control methods for common method bias. Adv. Psychol. Sci. 6, 942–950.

[ref36] Zhou X. L. Zhang M. Li B. Ma S. S. (2024). Cross-lagged analysis of social support, physical activity behavior, and family relationships among university students. Front. Psychol. 15:1439252. doi: 10.3389/fpsyg.2024.1439252, 39220389 PMC11363713

[ref37] Zhu Nan P. P. Zou Rong. (2015). The influence of socioeconomic status and social support on parent-child relationships in families with children with special needs. Chin. J. Spec. Educ. 9, 19–24.

